# Plasma Amino Acids Changes in Complex Regional Pain Syndrome

**DOI:** 10.1155/2013/742407

**Published:** 2013-11-04

**Authors:** Guillermo M. Alexander, Erin Reichenberger, B. Lee Peterlin, Marielle J. Perreault, John R. Grothusen, Robert J. Schwartzman

**Affiliations:** ^1^Department of Neurology, Drexel University College of Medicine, 245 North 15th Street, MS 423, Philadelphia, PA 19102, USA; ^2^Department of Neurology, Johns Hopkins University School of Medicine, 4940 Eastern Avenue, Baltimore, MD 21224, USA

## Abstract

Complex regional pain syndrome (CRPS) is a severe chronic pain condition that most often develops following trauma. Blood samples were collected from 220 individuals, 160 CRPS subjects, and 60 healthy pain-free controls. Plasma amino acid levels were compared and contrasted between groups. L-Aspartate, L-glutamate, and L-ornithine were significantly increased, whereas L-tryptophan and L-arginine were significantly decreased in CRPS subjects as compared to controls. In addition, the L-kynurenine to L-tryptophan ratio demonstrated a significant increase, whereas the global arginine bioavailability ratio (GABR) was significantly decreased in the CRPS subjects. The CRPS subjects demonstrated a significant correlation between overall pain and the plasma levels of L-glutamate and the L-kynurenine to L-tryptophan ratio. CRPS subjects also showed a correlation between the decrease in plasma L-tryptophan and disease duration. This study shows that CRPS subjects exhibit significant changes in plasma levels of amino acids involved in glutamate receptor activation and in amino acids associated with immune function as compared to healthy pain-free controls. A better understanding of the role plasma amino acids play in the pathophysiology of CRPS may lead to novel treatments for this crippling condition.

## 1. Introduction

Complex regional pain syndrome (CRPS), formerly reflex sympathetic dystrophy (RSD) or causalgia, is a severe chronic neuropathic pain condition [1, 2]. CRPS usually develops following trauma and is thought to involve both central and peripheral components of the neuraxis [1, 3]. The signs and symptoms of CRPS cluster into four distinct subgroups: (1) abnormalities in pain processing, (2) skin color and temperature changes, (3) edema, vasomotor, and sudomotor abnormalities, and (4) motor dysfunction and trophic changes [4]. Continuous pain is the most devastating of these symptoms and has been reported to spread and worsen over time, and it is usually disproportionate to the severity and duration of the inciting event [2].

CRPS may result from 5% of all nerve injuries [5, 6] and affects between 200,000 and 1.2 million Americans. In our pain clinic, CRPS demonstrates a 4 : 1 female to male preponderance and an average age of onset of 37 years old [3]. The incidence of CRPS reported 3 months following radial fractures (28%) is significantly higher than the incidence (7%) reported 1 year after the same fracture [7]. The majority of CRPS patients undergo resolution of their symptoms, often spontaneously, and in only a minority of patients does the disease become chronic [8]. However, after one year, most signs and symptoms become well developed and rarely resolve [3]. It remains unknown why most subjects undergo normal healing following an injury whereas others progress to a chronic painful condition with little or no chance of resolution.

Although the pathophysiology of CRPS is not completely understood, two mechanisms based largely on experimental animal models have wide support in the literature as contributors to its initiation and maintenance [2]. One mechanism is centered on neuronal sensitization and the other on neuroimmune interactions [9, 10]. Pain perception has been classically viewed as being mediated solely by neurons and most early studies of exaggerated pain conditions centered on peripheral and central neuronal sensitization as the driving mechanism [9, 11]. However, over the last decade, a vast number of studies support the notion of both glia and immune system involvement in chronic pain disorders including CRPS [12–16].

Following an injury, several immune mediated mechanisms can affect pain signaling systems. Mast cells, neutrophils, and macrophages are activated and recruited to the site of injury [17]. Disruption of the blood nerve barrier allows for the invasion of the nerve by fibroblasts, macrophages and Schwann cells [18]. These cells release proinflammatory cytokines and chemokines that have been implicated in the generation of neuropathic pain either via direct sensitization of nociceptors or indirectly by stimulating the release of agents that act on neurons and glia [16, 19, 20]. Microglia and astrocytes are the immunocompetent cells in the central nervous system (CNS) and are activated following tissue injury or inflammation [21]. Once activated, microglia and astrocytes release a number of substances known to excite pain transmission in neurons [22, 23]. Activated microglia and astrocytes have been shown to be both necessary and sufficient for enhanced nociception [16]. A number of studies have shown an altered cytokine profile in blood, cerebrospinal fluid, and blister fluid in patients with CRPS [24–27]. In addition, activation of both microglia and astrocytes has been reported in spinal cord autopsy tissue from a subject afflicted with CRPS [14].

Nerve injury and regional neural activation have also been implicated in the disruption of the blood-spinal cord barrier (BSCB) resulting in the influx of inflammatory mediators and the spinal recruitment of T cells and monocytes [28, 29]. T-Lymphocyte deficient mice demonstrate significantly less mechanical allodynia after nerve injury than wild-type animals [13]. The metabolism of L-tryptophan and L-arginine in myeloid cells has been reported to control T-cell proliferation, survival, and responses to antigens [30–33]. Deficiencies of L-arginine can contribute to endothelial cell and T-cell dysfunction as well as impairment of nitric oxide (NO) dependent signaling pathways in the CNS [34, 35].

The aim of this study was to extend previous reports demonstrating plasma amino acid alterations in neuropathic pain conditions such as fibromyalgia and CRPS type 1 [36–39]. Reductions in the plasma levels of both L-arginine and L-tryptophan have been reported in fibromyalgia patients [36, 37, 39]. Plasma alterations in amino acids known to be involved in N-methyl-D-aspartate (NMDA) receptor activation and the endothelium-dependent arginine-NO system have been reported in CRPS type 1 subjects [38]. However, there were several amino acids such as D-serine and L-kynurenine involved in these systems that were not evaluated in the aforementioned studies.

This study evaluated the plasma level of D-serine a co-agonist of the NMDA glutamate receptor [40, 41]. In addition to being expressed in CNS neurons [42, 43], glutamate receptors including the NMDA receptor are expressed at the peripheral processes of unmyelinated fibers [44]. Activation of these receptors has been demonstrated to contribute to hyperalgesia in animal models of neuropathic pain [44]. The NMDA glutamate receptor is also expressed in a variety of other cells including immune competent cells such as thymocytes, lymphocytes, and neutrophils [45, 46]. We evaluated L-kynurenine to explore a possible activation of the L-tryptophan to L-kynurenine pathway [40, 41, 47]. The activation of the L-kynurenine pathway by upregulation of indoleamine (2,3)-dioxygenase (IDO) has been linked to both pain and depression [48]. We also evaluated plasma levels of cortisol and neopterin in order to determine the relative involvement of tryptophan (2,3)-dioxygenase (TDO) and IDO in L-tryptophan metabolism. In addition, the previous study [38] evaluated plasma amino acids in CRPS type 1 subjects, this study included subjects afflicted with both CRPS type 1 and type 2 as well as healthy pain-free individuals as controls.

## 2. Methods

Men and nonpregnant women between the ages of 18 and 69 years old with a physician diagnosis of complex regional pain syndrome (CRPS) and healthy pain-free controls were recruited for this study. CRPS patients were recruited from the neurology pain clinic of Drexel University School of Medicine and met the clinical Budapest criteria for CRPS [49]. The CRPS subjects were classified as CRPS type 1 (no demonstrable nerve lesions) or type 2 (with demonstrable nerve lesions) [4]. Healthy pain-free control subjects were recruited from the community's general population. All subjects were enrolled after giving informed consent as approved by the Drexel University School of Medicine Institutional Review Boards (IRB). 

### 2.1. Patient Evaluation

All CRPS patients received a complete neurological examination and pain evaluation. Overall pain was determined on a 0–10 numerical rating scale (NRS) (0 was defined as no pain and a 10 was defined as the worst pain imaginable). The CRPS patients were evaluated and blood samples were obtained while taking their current medications. A list of these medications, other medical conditions reported by the subject, and blood values from a comprehensive metabolic panel and complete blood count with differential were obtained from their medical record. Additionally, the majority of the CRPS patients (108/160) underwent quantitative sensory testing (QST) to evaluate hot and cold detection thresholds as well as hot and cold pain thresholds. All clinical and pain evaluations were performed by the same investigator (RJS). All QST evaluations were also performed by one investigator (JRG). The detailed methods used for the CRPS patients neurologic and pain evaluation and the determination of thermal detection thresholds, thermal pain thresholds, motor function, and cutaneous temperature have been previously described [26, 50]. Medical history and self-reported values for height and weight were obtained from the normal healthy control subjects.

### 2.2. Blood Sampling

Venous non-fasted blood samples were collected into EDTA-coated (purple top) vacutainers between 8:00 a.m. and 12:00 noon. Blood samples were drawn from the cubital veins of subjects at rest. The plasma was separated by centrifugation (3000 ×g for 15 minutes at 4°C), split into 250 microliter aliquots, and stored at −70°C until assayed.

### 2.3. Determination of Plasma Amino Acids, Neopterin, and Cortisol

Plasma levels of L-alanine, L-arginine, L-asparagine, L-aspartate, L-citrulline, L-glutamate, L-glutamine, glycine, L-histidine, L-isoleucine, L-leucine, L-methionine, L-ornithine, L-phenylalanine, L-serine, D-serine, L-threonine, L-tyrosine, L-valine, taurine, and L-lysine were determined by high-performance liquid chromatography (HPLC) with fluorimetric detection using a modification of the method of Hashimoto et al. [51]. Plasma samples were deproteinated by ultrafiltration through a 10,000 molecular weight cutoff filter (Microcon Ultracel YM-10). The deproteinated plasma was precolumn derivatized with N-tert-butyloxycarbonyl-L-cysteine and O-phthaldialdehyde. The derivatized amino acids were analyzed using an octadecylsilane (C_18_) column (Agilent ZORBAX Eclipse Plus C18 4.6 × 150 mm, 3.5 um particle size) with a guard column (Agilent ZORBAX Eclipse Plus C18 4.6 × 50 mm, 3.5 um particle size). The column was operated at a constant flow rate of 0.8 mL/min. Mobile phase A was 0.1 M sodium acetate buffer (pH 6.0), containing 7% acetonitrile and 3% tetrahydrofuran, and mobile phase B was 0.1 M sodium acetate buffer containing 47% acetonitrile and 3% tetrahydrofuran. The separation of amino acid derivatives was performed using a gradient from mobile phase A to B over 70 minutes. The gradient program began isocratically at a mixture of 90% A, 10% B for 5 minutes and then a linear increase to 85% A, 15% B from 5 to 40 minutes, followed by a second linear increase to 20% A, 80% B from 40 to 70 minutes. This was followed by a linear decrease down to the initial mixture of 90% A, 10% B from 70 to 75 min. The column was maintained isocratically at this mixture for at least 2 min to reequilibrate the system before the next injection. The fluorescent amino acid derivative was detected using a Gilson model 121 fluorometer (Gilson Inc., Middleton, WI).

Plasma levels of L-tryptophan and L-kynurenine were determined by HPLC with electrochemical detection (HPLC-EC) using a modification of the method of Maneglier et al. [52]. Plasma samples were deproteinated with perchloric acid (100 *μ*L of plasma, 10 *μ*L of 35% perchloric acid, and 10 *μ*L of 450 *μ*M 3-O-methyl dopa (in 2 mM ascorbic acid) as an internal standard). The acidified plasma was vortexed for 30 seconds, allowed to sit at room temperature for two minutes, and then centrifuged for 5 minutes at 14,000 g at 4°C. The supernatant was diluted 1 : 10 with ultrapure water (25 *μ*L of supernatant plus 225 *μ*L of ultrapure water) and filtered through a 10,000 molecular weight cutoff filter (Microcon Ultracel YM-10). Twenty microliters of the filtrate was then injected into the HPLC system for analysis. HPLC was performed isocratically on an octadecylsilane (C18) column (Thermo Scientific Hypersil Gold 150 × 4, 3.0 *μ*m particle size) with a mobile phase of 4% acetonitrile in buffer (20 mM sodium phosphate, pH 4.5). Coulometric detection (Coulochem II 5200A, ESA, Inc., Bedford, MA) was accomplished with the guard cell at a potential of +0.45 volts, electrode number 1 at +0.30 volts and electrode number 2 at +0.875 volts. The internal standard was detected in electrode #1 whereas L-tryptophan and L-kynurenine were detected in electrode #2.

### 2.4. Determination of Plasma Neopterin and Cortisol

Plasma levels of neopterin and cortisol were determined by Enzyme-Linked Immunosorbent Assay (ELISA) using kits from IBL International (Hamburg, Germany) with sensitivities of 0.7 nmoles/L and 2.46 ng/mL, respectively. The assays were performed in duplicate according to the manufacturer's instructions.

### 2.5. Statistical Analysis

Differences in amino acid levels between the subjects and healthy controls were examined using Students *t*-test for independent groups. Correlation between parametric variables was determined by the evaluation of the Pearson product-moment correlation coefficient (*R*). For nonparametric variables, Spearman's rank correlation coefficient (rho) was evaluated. The data was considered significantly different if *P* < 0.05. Power analysis was used utilizing published values (mean and standard deviation) for the human plasma amino acids that were evaluated to estimate the number of subjects per group needed in order to observe a 33% change with a power of 80% and an alpha of 0.05. The number varied from 15 to 30 subjects per group (depending on the amino acid). For this study, we used all of the available samples in our CRPS blood bank, 160 CRPS subjects and 60 age- and gender-matched healthy controls, a number in excess of the sample size required by power analysis. Statistical calculations were accomplished with the aid of data analysis software, SYSTAT version 11 (SYSTAT Software Inc., Chicago, IL) and PASW Statistics version 18 (SPSS Inc. an IBM Company, Chicago, IL).

## 3. Results

### 3.1. Subject Demographics

A total of 220 subjects (160 CRPS and 60 controls) were enrolled in this study. One hundred and twenty-four of the CRPS subjects were type 1 and 36 type 2. The breakdown of the subject population by gender and race was as follows: the 60 subjects in the control group (48 females, 12 males) consisted of 47 Caucasians, 5 African Americans, 6 Asians, and 2 Hispanics, whereas the 160 subjects in the CRPS group (131 females, 29 males) consisted of 153 Caucasians, 4 African Americans, 1 Asian, and 2 Hispanics. The breakdown of the CRPS group by gender and race is consistent with the distribution observed in the CRPS population in our clinic and other clinics in the United States [3, 53]. The number of subjects in each group, their age, gender, and body mass index (BMI), as well as their CRPS type, duration of disease, and NRS pain score for the CRPS group, are tabulated in Table 1. There were no statistically significant differences (*P* > 0.05) in age, gender ratio, or BMI between the CRPS and the healthy control populations.

### 3.2. Pain and Quantitative Sensory Evaluation

The CRPS subjects reported NRS pain scores at the time the blood sample was taken that ranged from 1 to 10 with a median value of 7. However, most subjects reported pain greater than or equal to 4, with only 11 out of 160 subjects reporting pain levels less than 4 (no subject reported zero pain). On clinical examination, all CRPS subjects demonstrated abnormalities in pain processing with mechanoallodynia (static or dynamic) being the most frequently found symptom (142/160). Skin color and temperature changes were found in 80% of subjects, whereas edema, vasomotor, and sudomotor abnormalities were found in 83.8%. Motor dysfunction and trophic changes were found in 95% of the subjects with weakness being the most prevalent (145/160) motor finding.

One hundred and eight of the 160 CRPS subjects had quantitative thermal tests performed as part of their clinical evaluation. Alteration of cold or warm detection thresholds was determined using the normal cutoff limits reported by Yarnitsky and Sprecher [54]. Evidence of cold allodynia or heat hyperalgesia was determined using previously published values of cold and heat pain thresholds in normal control subjects [55, 56]. None of the subjects demonstrated low thresholds (hypersensitivity) to cold or warm stimuli. The majority of CRPS subjects (68/108) demonstrated elevated (hyposensitivity) thresholds to cold (49/108), heat (57/108), or both (39/108). Close to half of the CRPS patients (49/108) demonstrated thermal allodynia. Thirty eight patients showed cold allodynia, 38 demonstrated heat hyperalgesia and 27 demonstrated both. There were no significant differences (*P* > 0.05) in age, gender, BMI, overall pain, clinical signs and symptoms or quantitative thermal tests between the type 1 and type 2 CRPS subjects.

### 3.3. Plasma Levels of Amino Acids Neopterin and Cortisol

Plasma amino acid levels in both the CRPS and control groups are listed in Table 2. The plasma levels of L-aspartate, L-glutamate, and L-ornithine were significantly increased (*P* < 0.005) whereas L-tryptophan and L-arginine were significantly decreased (*P* < 0.05) in the CRPS group as compared to healthy controls. All probabilities were adjusted for multiple testing (Bonferroni). In addition, the L-kynurenine to L-tryptophan ratio (an index of indoleamine (2,3)-dioxygenase (IDO) activity) demonstrated a significant (*P* < 0.001) increase between the CRPS group and healthy controls, whereas the global arginine bioavailability ratio (GABR) (defined as L-arginine/[L-ornithine + L-citrulline]) was significantly (*P* < 0.0001) decreased in the CRPS subjects (Table 3).

Plasma levels of neopterin and cortisol in both the CRPS and control groups are listed in Table 3.

There was a significant (*P* < 0.05) increase in plasma neopterin, a biomarker of immune activation [47, 57], in the CRPS group as compared to healthy controls. However, there was no significant change (*P* > 0.05) in plasma cortisol levels between CRPS and healthy control subjects.

For all amino acids, neopterin and cortisol, there was no significant sex differences (*P* > 0.05) in plasma levels within the healthy control or CRPS patient groups. There was also no significant difference (*P* > 0.05) in plasma levels of all analytes between the type 1 and type 2 CRPS subjects. Some amino acids and neopterin demonstrated a significant (*P* < 0.05) correlation between their plasma levels and BMI (Table 4) or age (Table 5). However, when corrected for BMI, age, or both, the statistical significant differences between CRPS subjects and controls shown by these analytes remained unchanged. 

**Figure 1:**
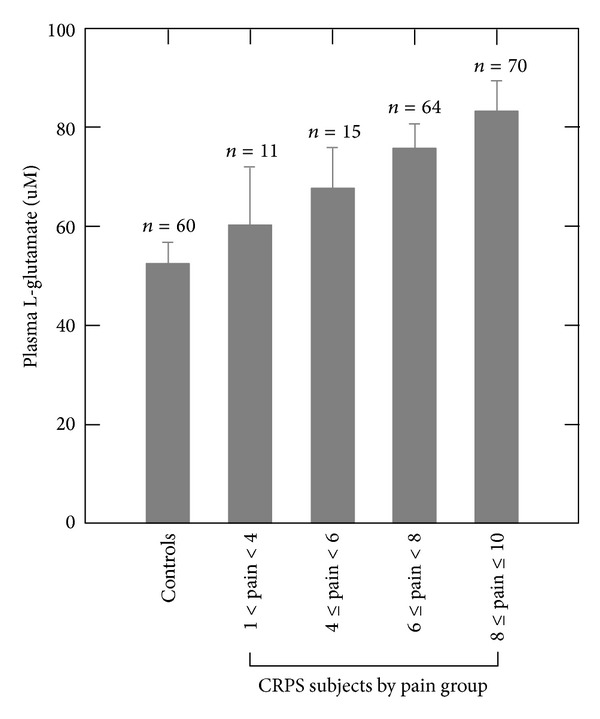
Plasma levels of L-glutamate (mean ± standard deviation) in healthy pain-free controls and CRPS subjects grouped by increasing overall NRS pain values. The plasma level of L-glutamate in the CRPS subjects demonstrated a significant positive correlation (rho = 0.156, *P* = 0.050) with overall pain.

**Figure 2:**
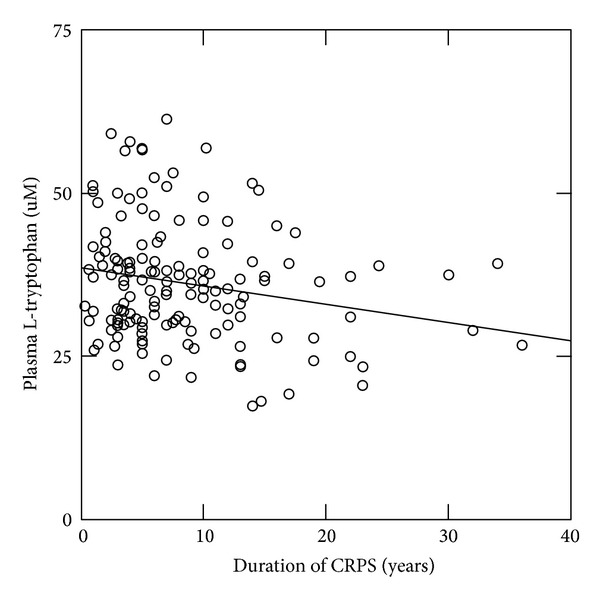
Plasma levels of L-tryptophan in the CRPS subjects versus duration of CRPS (years between initiating event and blood draw). The plasma level of L-tryptophan in the CRPS subjects demonstrated a significant negative correlation (*R* = 0.211, *P* = 0.0105) with duration of CRPS.

The CRPS subjects in this study demonstrated no significant correlation (*P* > 0.05) between the plasma level of any analyte (amino acids, neopterin, or cortisol) and age at onset of symptoms, cold allodynia, heat hyperalgesia, or cold temperature detection threshold. However, the CRPS subjects demonstrated a significant correlation between overall pain and the plasma levels of L-glutamate (rho = 0.156, *P* = 0.050) (Figure 1), the L-kynurenine to L-tryptophan ratio (rho = 0.166, *P* = 0.043), and neopterin (rho = 0.175, *P* = 0.036). In addition, the plasma levels of L-glutamate showed a significant correlation with warm detection threshold (*R* = 0.252, *P* = 0.0089) and L-tryptophan with duration of CRPS (*R* = 0.211, *P* = 0.0105) (Figure 2).

The most frequently prescribed medications in our CRPS group were narcotics, antidepressants, antiepileptics, and antianxiolytics. There was no significant difference (*P* > 0.05) in plasma analyte levels between CRPS subjects taking these medications and those who were not. There was also no significant difference (*P* > 0.05) in plasma analyte levels in the CRPS subjects when grouped by the number of medications they were taking. The CRPS patients in this study reported a number of other diagnosed conditions. The following conditions (including the percent afflicted if greater than 5%) were reported by the study subjects: depression (66.9%), headaches (65.6%), generalized anxiety disorder (53.1%), gastroesophageal reflux disease (GERD) (33.3%), hypertension (31.3%), high cholesterol (26.3%), irritable bowel syndrome (22.5%), radiculopathies (23.8%), cardiovascular disease (17.5%), thyroid problems (15.6%), arthritis (13.8%), and disk disease (11.9%). There was no significant difference (*P* > 0.05) in plasma analyte levels between CRPS subjects exhibiting any of these conditions and those who did not.

## 4. Discussion

This study demonstrated differences in the plasma level of a number of amino acids in subjects afflicted with CRPS as compared to pain-free healthy controls. There were three principal findings: (1) significant increases (*P* < 0.005) in plasma L-glutamate and L-aspartate with no significant (*P* > 0.05) difference in plasma levels of glycine and D-serine (coagonists at the NMDA receptor); (2) significant (*P* < 0.05) but modest decrease in plasma L-tryptophan along with a significant (*P* < 0.001) increase in the L-kynurenine to L-tryptophan ratio; and (3) a significant (*P* < 0.0001) decrease in plasma L-arginine with a concomitant significant (*P* < 0.0001) decrease in its bioavailability (GABR).

The alterations in the plasma concentrations of these amino acids have functional consequences in the pathophysiology of CRPS. In addition to being expressed in CNS neurons [42, 43], glutamate receptors including the NMDA receptor are expressed at the peripheral processes of unmyelinated fibers [44]. Activation of these receptors has been demonstrated to contribute to hyperalgesia in animal models of neuropathic pain [44]. Significant increase in plasma L-glutamate has been previously reported in CRPS type 1 subjects [38].

The metabolism of L-tryptophan and L-arginine in myeloid cells has been reported to control T-cell proliferation, survival, and responses to antigens [30–33]. In animal models, T cells play an important role in the development of neuropathic pain. Peripheral nerve injury has been shown to increase the infiltration of T cells in the adult mouse spinal cord [13]. T-lymphocyte deficient mice demonstrate significantly less mechanical allodynia after nerve injury than wild-type animals [13]. Reductions in the plasma levels of both of these amino acids have been reported in fibromyalgia patients [36, 37, 39].

### 4.1. L-Tryptophan and the L-Kynurenine to L-Tryptophan Ratio

L-Tryptophan is an essential amino acid and as such it cannot be synthesized and must be supplied in the diet. L-Tryptophan is metabolized by tryptophan hydroxylase, the rate-limiting step, leading to the synthesis of serotonin [58] and by enzymes in the kynurenine pathway [47]. Two enzymes, tryptophan (2,3)-dioxygenase (TDO) and indoleamine (2,3)-dioxygenase (IDO), catabolize tryptophan via the kynurenine pathway [47]. IDO is expressed by a variety of cells and is inducible preferentially by Th1-type cytokines whereas TDO is localized to the liver and is upregulated by corticosteroids [47].

In addition to the decrease in the plasma levels of L-tryptophan, the CRPS subjects demonstrated a significant (*P* < 0.05) increase in the L-kynurenine to L-tryptophan ratio (kyn/trp) an index of activation of the kynurenine pathway [59]. The activation of IDO has been linked to both pain and depression [48, 60]. Activated IDO is indicated when kyn/trp correlates with an immune activation parameter such as plasma neopterin whose concentration has been shown to be a sensitive biomarker of Th1-type immune activation in humans [47, 57]. There was a significant correlation between kyn/trp and plasma neopterin levels in the CRPS subjects (*R* = 0.385, *P* < 0.0001) whereas kyn/trp did not correlate with plasma cortisol. The fact that the CRPS subjects demonstrated significant (*P* < 0.05) increases in both kyn/trp and neopterin as compared to controls strongly suggests immune activation and IDO upregulation in CRPS.

### 4.2. L-Arginine and the Global Arginine Bioavailability Ratio

The modulation of T-cell function by monocytes and granulocytes can also be accomplished by manipulating the metabolism of L-arginine [30, 61]. L-Arginine is classified as a semiessential amino acid given that the body usually produces enough of it from L-citrulline by the kidney; L-citrulline, in turn, is produced from glutamine in the intestine [62]. Two enzymes are involved in L-arginine metabolism: (1) the nitric oxide synthases (NOS1, NOS2, and NOS3) that generate nitric oxide (NO) and L-citrulline and (2) the arginases (Arg1 and Arg2) that produce urea and L-ornithine [61, 63, 64]. Given that the metabolisms of L-arginine, L-citrulline, and L-ornithine are interconnected, the global arginine bioavailability ratio (GABR) is a better index of dysregulation of L-arginine metabolism compared to systemic arginine levels [34]. GABR was found to be significantly decreased (*P* < 0.00001) in the CRPS subjects in this study as compared to controls.

Deficiencies of L-arginine can contribute to endothelial cell and T-cell dysfunction as well as dysfunction of NO dependent signaling pathways in the CNS [34, 35]. A number of animal studies have shown that inhibition of NO synthesis from L-arginine can considerably reduce both inflammatory and neuropathic pain [35, 65]. However, there are conflicting results showing both pronociceptive and antinociceptive effects of NO. Some studies have shown that, in animal models of inflammatory and neuropathic pain, local inhibition of NO synthesis in the spinal cord leads to a reduction of nociceptive behavior [66, 67]. Other studies report that the intrathecal administration of the NO precursor L-arginine reduced dorsal horn neuronal activity in response to a noxious stimulus and an increased mechanical threshold for tail withdrawal [68, 69]. The effect of decreased L-arginine bioavailability on NO synthesis and its consequence to the pathophysiology of CRPS need further evaluation.

### 4.3. Amino Acids Involved in NMDA Receptor Activation

This study also found that, in CRPS subjects, amino acids involved in NMDA receptor activation such as L-glutamate and L-aspartate demonstrated significant (*P* < 0.005) increases in their plasma levels as compared to healthy pain-free controls. However, the plasma levels of D-serine and glycine, the coagonists of the NMDA glutamate receptor [40, 41], although slightly increased, did not differ significantly (*P* > 0.05) from controls.

Although some investigators postulate that plasma L-glutamate levels reflect levels of L-glutamate in brain [70], the current consensus in that L-glutamate does not readily cross the blood-brain barrier [71]. Under normal conditions, as in the periphery, brain L-glutamate is derived from local synthesis from other amino acids and citric acid cycle intermediates [72, 73]. It is therefore unlikely that elevated plasma L-glutamate would have an effect on central NMDA receptors. However, in addition to being expressed in CNS neurons where it mediates calcium influx in response to synaptic activity [42, 43], the NMDA glutamate receptor is expressed in the peripheral processes of unmyelinated fibers where its activation contributes to hyperalgesia in animal models of neuropathic pain [44]. Elevated plasma levels of L-glutamate can also increase the activation of peripheral metabotropic glutamate receptors which have been liked to both nociception and immune modulation [44, 74, 75]. Both glutamate receptor activation and neuroimmune modulation have been associated with the pathophysiology of CRPS [9, 10].

The NMDA glutamate receptor is also expressed in a variety of nonneuronal cells such as astrocytes and oligodendrocytes where they are thought to mediate neuron to glia signaling [76, 77]; in erythrocytes, mediating calcium influx that activates nitric oxide synthase which produces nitric oxide in the presence of L-arginine [78]; in neuroepithelial cells where they are thought to control glutamate transporters and the neuroepithelial barrier [79]; and in immune cells where they play a role in modulating immune function [45]. The increased plasma levels of L-glutamate and L-aspartate observed in the CRPS subjects in this study may cause the activation of peripheral NMDA receptors. The efficacy of NMDA receptor antagonists in the treatment of CRPS [80–83] may be due in part to NMDA receptor inhibition at peripheral sites. 

Changes in plasma amino acid levels have been previously reported in type 1 CRPS [38]. The study demonstrated increased plasma levels of glutamate, arginine, serine, taurine, glycine, and phenylalanine with decreased plasma levels of methionine (amino acid results from their study are listed without the L or D prefix given that their methods did not allow for the separation of L from D amino acids) [38]. Other than the increase in plasma L-glutamate, there was little agreement between our findings and those of these investigators and the 47% increase in plasma L-glutamate reported in our study was much smaller than the 574% increase reported in theirs [38]. Both studies collected blood from nonfasted subjects and the plasma amino acid values for our control subjects did not differ from the control values reported in their study, suggesting that the variance between the two studies were not due to the methodological differences. Further studies are required in order to resolve the differing results between these studies.

### 4.4. Study Limitations

There are several limitations of this study. The study subjects were not fasted prior to obtaining blood samples. However, there was little change in the plasma levels of the essential amino acids (amino acids that cannot be synthesized and therefore must be supplied in the diet) between CRPS subjects and controls. Of the eight essential amino acids evaluated in this study, only tryptophan demonstrated a significant (although modest) difference between CRPS and control subjects. These facts do not support the suggestion that the amino acid changes reported in this study were diet related. Depression questionnaires (such as the Becks depression inventory) were not administered to evaluate the CRPS subject's severity of depression and its contribution to altered plasma amino acids. However, given that the plasma amino acid levels did not differ between CRPS subjects with (*n* = 107) and without (*n* = 53) depression, the authors feel that this limitation does not negate our findings.

## 5. Conclusion

The results of this study suggest that the increase in plasma L-glutamate may affect peripheral glutamate receptors involved in nociception and immune modulation, that the decrease in plasma L-tryptophan is most likely due to upregulation of IDO, whereas the reduction of plasma L-arginine is likely due to increased arginase activity. However, upregulation of the nitric oxide synthases is also a possibility. Our results also support that, in the CRPS subjects, increases in plasma L-glutamate correlate with the degree of pain whereas decreased plasma L-tryptophan is associated with disease duration. Additional studies are needed to discern the mechanisms by which plasma amino acids are altered as well as the role that these alterations play in the pathophysiology of CRPS. A better understanding of these mechanisms may lead to novel treatments for this crippling condition.

## Figures and Tables

**Table 1:** Subject demographics.

Subject group	*N*	Age	M/F	BMI	Type	Duration	NRS
		Years (range)			1/2	Years (range)	(0–10)
Controls	60	40.7 (23–68)	12/48	26.2 ± 0.8	N/A	N/A	N/A
CRPS	160	43.7 (18–68)	29/131	26.9 ± 0.5	124/36	8.6 (0.5–36)	7 (1–10)

This table lists the two groups, the number of subjects (*N*) in each group, their average age and range in years, the gender ratio (males/females), and the body mass index (BMI, mean ± standard error). In addition, for the CRPS patients, it lists the number of CRPS type 1 and 2 patients, the mean and range of the disease duration (years between initiating event and blood draw) and the median and range of the NRS pain score. There were no significant differences in age (*P* = 0.12), BMI (*P* = 0.38), or gender makeup (*P* = 0.75) between CRPS subjects and controls.

**Table 2:** Plasma amino acid levels.

Amino acid	Controls (*n* = 60)	CRPS (*n* = 160)	*P*
	(umoles/L)	(umoles/L)	
L-Aspartate	7.47 ± 0.36	9.27 ± 0.30	**0.00473**
L-Glutamate	52.51 ± 4.29	77.22 ± 3.52	**0.00057**
L-Asparagine	51.91 ± 1.69	47.59 ± 0.93	
L-Serine	107.75 ± 3.54	100.16 ± 2.23	
L-Glutamine	572.73 ± 13.2	579.40 ± 9.74	
D-Serine	1.94 ± 0.07	2.06 ± 0.05	
L-Citrulline	28.17 ± 1.20	30.58 ± 0.84	
L-Threonine	135.07 ± 5.04	133.34 ± 3.08	
Glycine	302.99 ± 12.6	318.87 ± 9.31	
L-Histidine	65.88 ± 2.28	68.07 ± 1.91	
L-Arginine	67.49 ± 3.33	48.82 ± 1.59	**0.000001**
L-Alanine	426.06 ± 14.2	447.11 ± 11.1	
Taurine	67.46 ± 2.61	72.87 ± 2.29	
L-Tyrosine	80.72 ± 2.82	77.39 ± 1.77	
L-Valine	260.67 ± 8.54	243.64 ± 5.46	
L-Methionine	23.81 ± 0.87	20.76 ± 0.50	
L-Isoleucine	74.93 ± 3.51	66.89 ± 1.90	
L-Tryptophan	41.91 ± 1.25	36.96 ± 0.82	**0.04210**
L-Phenylalanine	48.23 ± 1.45	46.53 ± 0.92	
L-Leucine	122.71 ± 4.70	108.53 ± 2.57	
L-Ornithine	70.29 ± 3.53	94.38 ± 3.12	**0.00002**
L-Lysine	160.89 ± 5.76	155.57 ± 3.37	
L-Kynurenine	1.79 ± 0.07	1.80 ± 0.06	

This table lists the plasma amino acid levels in CRPS and healthy control subjects. All values are given in micromoles per liter ± the standard error of the mean. Significant difference in plasma amino acid levels between the control and CRPS groups was determined with the Student's *t*-test adjusted for multiple testing (Bonferroni). Bonferroni adjusted probability values (the *P* value from the Student's *t*-test multiplied by 23, the number of amino acids evaluated) less than 0.05 are highlighted in bold.

**Table 3:** Plasma neopterin, cortisol, global arginine bioavailability ratio, and L-kynurenine to L-tryptophan ratio.

Analyte	Controls	CRPS	*P*
	(*n* = 60)	(*n* = 160)	
GABR	0.727 ± 0.037	0.437 ± 0.018	1.0 × 10^−09^
L-Kyn/L-Trp	0.043 ± 0.001	0.050 ± 0.001	**0.00097**
Neopterin (nM)	5.97 ± 0.22	6.76 ± 0.20	**0.01245**
Cortisol (ng/mL)	92.51 ± 7.81	87.08 ± 4.00	0.56200

This table lists the global arginine bioavailability ratio (GABR) (defined as plasma L-arginine/(L-ornithine + L-citrulline), the plasma L-kynurenine to L-tryptophan ratio, and the plasma levels of neopterin and cortisol in CRPS and healthy control subjects. Values are given as the mean ± the standard error of the mean. Significant difference between the control and CRPS groups was determined with the Student's *t*-test. Statistically significant probability values (*P* < 0.05) are highlighted in bold.

**Table 4:** Correlations between plasma amino acids and BMI.

Amino acid	Controls linear regression	CRPS linear regression
L-Asp	*R* = 0.410, *P* = 0.00115	
L-Glu	*R* = 0.445, *P* = 0.00037	*R* = 0.340, *P* = 0.00001
L-Ser		*R* = 0.216, *P* = 0.00616
L-Ala		*R* = 0.301, *P* = 0.00011
L-Tyr		*R* = 0.228, *P* = 0.00380
L-Val	*R* = 0.314, *P* = 0.01460	
L-Leu	*R* = 0.292, *P* = 0.02375	
L-Kyn		*R* = 0.186, *P* = 0.02204

This table list Pearson's correlation coefficient (*R*) and probability (*P*) for amino acids that demonstrated significant (*P* < 0.05) correlation between their plasma level and BMI. The amino acid L-serine demonstrated an inverse correlation with BMI, whereas all other amino acids listed in this table demonstrated a positive linear correlation.

**Table 5:** Correlations between plasma amino acids and age.

Amino acid	Controls linear regression	CRPS linear regression
L-Gln	*R* = 0.298, *P* = 0.02090	
L-Cit	*R* = 0.484, *P* = 0.00009	*R* = 0.234, *P* = 0.00293
L-Arg	*R* = 0.278, *P* = 0.03132	
L-Tyr		*R* = 0.288, *P* = 0.00225
L-Val		*R* = 0.166, *P* = 0.03647
L-Orn	*R* = 0.311, *P* = 0.01760	*R* = 0.160, *P* = 0.04290

This table list Pearson's correlation coefficient (*R*) and probability (*P*) for amino acids that demonstrated significant (*P* < 0.05) correlation between their plasma level and the subjects age. All amino acids listed in this table demonstrated positive linear correlation with age.

## References

[1] Jänig W, Baron R (2003). Complex regional pain syndrome: mystery explained?. *The Lancet Neurology*.

[2] Schwartzman RJ, Alexander GM, Grothusen J (2006). Pathophysiology of complex regional pain syndrome. *Expert Review of Neurotherapeutics*.

[3] Schwartzman RJ, Erwin KL, Alexander GM (2009). The natural history of complex regional pain syndrome. *Clinical Journal of Pain*.

[4] Harden RN, Bruehl S, Stanton-Hicks M, Wilson PR (2007). Proposed new diagnostic criteria for complex regional pain syndrome. *Pain Medicine*.

[5] Loeser JD (2001). *Bonica's the Management of Pain*.

[6] Plewes LW (1956). Sudek's atrophy in the hands. *The Journal of Bone & Joint Surgery*.

[7] Bickerstaff DR, Kanis JA (1994). Algodystrophy: an under-recognized complication of minor trauma. *British Journal of Rheumatology*.

[8] Sandroni P, Benrud-Larson LM, McClelland RL, Low PA (2003). Complex regional pain syndrome type I: incidence and prevalence in Olmsted county, a population-based study. *Pain*.

[9] Costigan M, Scholz J, Woolf CJ (2009). Neuropathic pain: a maladaptive response of the nervous system to damage. *Annual Review of Neuroscience*.

[10] Watkins LR, Maier SF (2005). Immune regulation of central nervous system functions: from sickness responses to pathological pain. *Journal of Internal Medicine*.

[11] Woolf CJ, Salter MW (2000). Neuronal plasticity: increasing the gain in pain. *Science*.

[12] Austin PJ, Moalem-Taylor G (2010). The neuro-immune balance in neuropathic pain: involvement of inflammatory immune cells, immune-like glial cells and cytokines. *Journal of Neuroimmunology*.

[13] Costigan M, Moss A, Latremoliere A (2009). T-cell infiltration and signaling in the adult dorsal spinal cord is a major contributor to neuropathic pain-like hypersensitivity. *Journal of Neuroscience*.

[14] del Valle L, Schwartzman RJ, Alexander G (2009). Spinal cord histopathological alterations in a patient with longstanding complex regional pain syndrome. *Brain, Behavior, and Immunity*.

[15] Goebel A, Blaes F (2013). Complex regional pain syndrome, prototype of a novel kind of autoimmune disease. *Autoimmunity Reviews*.

[16] Watkins LR, Maier SF (2003). Glia: a novel drug discovery target for clinical pain. *Nature Reviews Drug Discovery*.

[17] Marchand F, Perretti M, McMahon SB (2005). Role of the immune system in chronic pain. *Nature Reviews Neuroscience*.

[18] McMahon SB, Cafferty WBJ, Marchand F (2005). Immune and glial cell factors as pain mediators and modulators. *Experimental Neurology*.

[19] Abbadie C (2005). Chemokines, chemokine receptors and pain. *Trends in Immunology*.

[20] DeLeo JA, Colburn RW, Nichols M, Malhotra A (1996). Interleukin-6-mediated hyperalgesia/allodynia and increased spinal IL-6 expression in a rat mononeuropathy model. *Journal of Interferon and Cytokine Research*.

[21] Tsuda M, Inoue K, Salter MW (2005). Neuropathic pain and spinal microglia: a big problem from molecules in ‘small’ glia. *Trends in Neurosciences*.

[22] Kreutzberg GW (1996). Microglia: a sensor for pathological events in the CNS. *Trends in Neurosciences*.

[23] Watkins LR, Milligan ED, Maier SF (2001). Glial activation: a driving force for pathological pain. *Trends in Neurosciences*.

[24] Alexander GM, van Rijn MA, van Hilten JJ, Perreault MJ, Schwartzman RJ (2005). Changes in cerebrospinal fluid levels of pro-inflammatory cytokines in CRPS. *Pain*.

[25] Alexander GM, Perreault MJ, Reichenberger ER, Schwartzman RJ (2007). Changes in immune and glial markers in the CSF of patients with complex regional pain syndrome. *Brain, Behavior, and Immunity*.

[26] Alexander GM, Peterlin BL, Perreault MJ, Grothusen JR, Schwartzman RJ (2012). Changes in plasma cytokines and their soluble receptors in complex regional pain syndrome. *Journal of Pain*.

[27] Huygen FJPM, de Bruijn AGJ, de Bruin MT, George Groeneweg J, Klein J, Zijlstra FJ (2002). Evidence for local inflammation in complex regional pain syndrome type 1. *Mediators of Inflammation*.

[28] Arima Y, Harada M, Kamimura D (2012). Regional neural activation defines a gateway for autoreactive T cells to cross the blood-brain barrier. *Cell*.

[29] Echeverry S, Shi XQ, Rivest S, Zhang J (2011). Peripheral nerve injury alters blood-spinal cord barrier functional and molecular integrity through a selective inflammatory pathway. *Journal of Neuroscience*.

[30] Bronte V, Zanovello P (2005). Regulation of immune responses by L-arginine metabolism. *Nature Reviews Immunology*.

[31] Grohmann U, Fallarino F, Puccetti P (2003). Tolerance, DCs and tryptophan: much ado about IDO. *Trends in Immunology*.

[32] Hwang SL, Chung NP-Y, Chan JK-Y, Lin C-LS (2005). Indoleamine 2,3-dioxygenase (IDO) is essential for dendritic cell activation and chemotactic responsiveness to chemokines. *Cell Research*.

[33] Ploder M, Spittler A, Kurz K (2010). Accelerated tryptophan degradation predicts poor survival in trauma and sepsis patients. *International Journal of Tryptophan Research*.

[34] Morris SM (2012). Arginases and arginine deficiency syndromes. *Current Opinion in Clinical Nutrition & Metabolic Care*.

[35] Schmidtko A, Tegeder I, Geisslinger G (2009). No NO, no pain? The role of nitric oxide and cGMP in spinal pain processing. *Trends in Neurosciences*.

[36] Bazzichi L, Palego L, Giannaccini G (2009). Altered amino acid homeostasis in subjects affected by fibromyalgia. *Clinical Biochemistry*.

[37] Moldofsky H, Warsh JJ (1978). Plasma tryptophan and musculoskeletal pain in non-articular rheumatism (“fibrositis syndrome”). *Pain*.

[38] Wesseldijk F, Fekkes D, Huygen FJPM, van de Heide-Mulder M, Zijlstra FJ (2008). Increased plasma glutamate, glycine, and arginine levels in complex regional pain syndrome type 1. *Acta Anaesthesiologica Scandinavica*.

[39] Yunus NB, Dailey JW, Aldag JC, Masi AT, Jobe PC (1992). Plasma tryptophan and other amino acids in primary fibromyalgia: a controlled study. *Journal of Rheumatology*.

[40] Snyder SH, Ferris CD (2000). Novel neurotransmitters and their neuropsychiatric relevance. *The American Journal of Psychiatry*.

[41] Wolosker H (2006). D-serine regulation of NMDA receptor activity. *Science’s STKE*.

[42] Sobczyk A, Svoboda K (2007). Activity-dependent plasticity of the NMDA-receptor fractional Ca^2+^ current. *Neuron*.

[43] Zito K, Scheuss V, Knott G, Hill T, Svoboda K (2009). Rapid functional maturation of nascent dendritic spines. *Neuron*.

[44] Jang JH, Kim D-W, Sang Nam T, Se Paik K, Leem JW (2004). Peripheral glutamate receptors contribute to mechanical hyperalgesia in a neuropathic pain model of the rat. *Neuroscience*.

[45] Boldyrev AA, Bryushkova EA, Vladychenskaya EA (2012). NMDA receptors in immune competent cells. *Biochemistry*.

[46] Mashkina AP, Cizkova D, Vanicky I, Boldyrev AA (2010). NMDA receptors are expressed in lymphocytes activated both in vitro and in vivo. *Cellular and Molecular Neurobiology*.

[47] Schröcksnadel K, Wirleitner B, Winkler C, Fuchs D (2006). Monitoring tryptophan metabolism in chronic immune activation. *Clinica Chimica Acta*.

[48] Kim H, Chen L, Lim G (2012). Brain indoleamine 2, 3-dioxygenase contributes to the comorbidity of pain and depression. *Journal of Clinical Investigation*.

[49] Harden RN, Bruehl S, Perez RSGM (2010). Validation of proposed diagnostic criteria (the “Budapest Criteria”) for complex regional pain syndrome. *Pain*.

[50] Grothusen JR, Alexander GM, Erwin K, Schwartzman RJ Thermal pain in complex regional pain syndrome type I.

[51] Hashimoto A, Nishikawa T, Oka T, Takahashi K, Hayashi T (1992). Determination of free amino acid enantiomers in rat brain and serum by high-performance liquid chromatography after derivatization with N-*tert*.-butyloxycarbonyl-L-cysteine and *o*-phthaldialdehyde. *Journal of Chromatography B*.

[52] Maneglier B, Rogez-Kreuz C, Cordonnier P (2004). Simultaneous measurement of kynurenine and tryptophan in human plasma and supernatants of cultured human cells by HPLC with coulometric detection. *Clinical Chemistry*.

[53] Sharma A, Agarwal S, Broatch J, Raja SN (2009). A web-based cross-sectional epidemiological survey of complex regional pain syndrome. *Regional Anesthesia and Pain Medicine*.

[54] Yarnitsky D, Sprecher E (1994). Thermal testing: normative data and repeatability for various test algorithms. *Journal of the Neurological Sciences*.

[55] Wasner GL, Brock JA (2008). Determinants of thermal pain thresholds in normal subjects. *Clinical Neurophysiology*.

[56] Yarnitsky D, Sprecher E, Zaslansky R, Hemli JA (1995). Heat pain thresholds: normative data and repeatability. *Pain*.

[57] Murr C, Gerlach D, Widner B, Dierich MP, Fuchs D (2001). Neopterin production and tryptophan degradation in humans infected by Streptococcus pyogenes. *Medical Microbiology and Immunology*.

[58] Grahame-Smith DG (1967). The biosynthesis of 5-hydroxytryptamine in brain. *Biochemical Journal*.

[59] Fuchs D, Möller AA, Reibnegger G, Stöckle E, Werner ER, Wachter H (1990). Decreased serum tryptophan in patients with HIV-1 infection correlates with increased serum neopterin and with neurologic/psychiatric symptoms. *Journal of Acquired Immune Deficiency Syndromes*.

[60] Bell C, Abrams J, Nutt D (2001). Tryptophan depletion and its implications for psychiatry. *British Journal of Psychiatry*.

[61] Bronte V, Serafini P, Mazzoni A, Segal DM, Zanovello P (2003). L-arginine metabolism in myeloid cells controls T-lymphocyte functions. *Trends in Immunology*.

[62] Windmueller HG, Spaeth AE (1981). Source and fate of circulating citrulline. *The American Journal of Physiology—Endocrinology and Metabolism*.

[63] Bogdan C (2001). Nitric oxide and the immune response. *Nature Immunology*.

[64] Wu G, Morris SM (1998). Arginine metabolism: nitric oxide and beyond. *Biochemical Journal*.

[65] Levy D, Zochodne DW (2004). NO pain: potential roles of nitric oxide in neuropathic pain. *Pain Practice*.

[66] Luo ZD, Cizkova D (2000). The role of nitric oxide in nociception. *Current Review of Pain*.

[67] Meller ST, Gebhart GF (1993). Nitric oxide (NO) and nociceptive processing in the spinal cord. *Pain*.

[68] Haley JE, Dickenson AH, Schachter M (1992). Electrophysiological evidence for a role of nitric oxide in prolonged chemical nociception in the rat. *Neuropharmacology*.

[69] Zhuo M, Meller ST, Gebhart GF (1993). Endogenous nitric oxide is required for tonic cholinergic inhibition of spinal mechanical transmission. *Pain*.

[70] Shimmura C, Suda S, Tsuchiya KJ (2011). Alteration of plasma glutamate and glutamine levels in children with high-functioning autism. *PLoS ONE*.

[71] Sheldon AL, Robinson MB (2007). The role of glutamate transporters in neurodegenerative diseases and potential opportunities for intervention. *Neurochemistry International*.

[72] Reeds PJ, Burrin DG, Stoll B, Jahoor F (2000). Intestinal glutamate metabolism. *Journal of Nutrition*.

[73] Smith QR (2000). Transport of glutamate and other amino acids at the blood-brain barrier. *Journal of Nutrition*.

[74] Julio-Pieper M, Flor PJ, Dinan TG, Cryan JF (2011). Exciting times beyond the brain: metabotropic glutamate receptors in peripheral and non-neural tissues. *Pharmacological Reviews*.

[75] Karim F, Bhave G, Gereau RW (2001). Metabotropic glutamate receptors on peripheral sensory neuron terminals as targets for the development of novel analgesics. *Molecular Psychiatry*.

[76] Lalo U, Pankratov Y, Kirchhoff F, North RA, Verkhratsky A (2006). NMDA receptors mediate neuron-to-glia signaling in mouse cortical astrocytes. *Journal of Neuroscience*.

[77] Verkhratsky A, Kirchhoff F (2007). NMDA receptors in glia. *Neuroscientist*.

[78] Makhro A, Wang J, Vogel J (2010). Functional NMDA receptors in rat erythrocytes. *The American Journal of Physiology—Cell Physiology*.

[79] Sharp CD, Fowler M, Jackson TH (2003). Human neuroepithelial cells express NMDA receptors. *BMC Neuroscience*.

[80] Correll GE, Maleki J, Gracely EJ, Muir JJ, Harbut RE (2004). Subanesthetic ketamine infusion therapy: a retrospective analysis of a novel therapeutic approach to complex regional pain syndrome. *Pain Medicine*.

[81] Kiefer RT, Rohr P, Ploppa A (2008). Efficacy of ketamine in anesthetic dosage for the treatment of refractory complex regional pain syndrome: an open-label phase II study. *Pain Medicine*.

[82] Schwartzman RJ, Alexander GM, Grothusen JR, Paylor T, Reichenberger E, Perreault M (2009). Outpatient intravenous ketamine for the treatment of complex regional pain syndrome: a double-blind placebo controlled study. *Pain*.

[83] Sigtermans MJ, van Hilten JJ, Bauer MCR (2009). Ketamine produces effective and long-term pain relief in patients with complex regional pain syndrome type 1. *Pain*.

